# Cholesterol, Oxysterols and LXRs in Breast Cancer Pathophysiology

**DOI:** 10.3390/ijms21041356

**Published:** 2020-02-17

**Authors:** Hassan Nazih, Jean Marie Bard

**Affiliations:** 1 Laboratoire de Biochimie Générale et Appliquée, UFR de Pharmacie, MMS-EA 2160-Mer Molécules Santé, IUML-Institut Universitaire Mer et Littoral-FR3473 CNRS, Université de Nantes, 44035 Nantes, France; jean-marie.bard@univ-nantes.fr; 2 Institut de Cancérologie de l’Ouest, Boulevard Jacques Monod, 44805 Saint-Herblain CEDEX, France

**Keywords:** cholesterol, oxysterols, breast cancer, LXRs

## Abstract

Breast cancer is the most frequent cancer among women. In 2018, it is estimated that 627,000 women died from breast cancer. This is approximately 15% of all cancer deaths among women (WHO 2018). Breast cancer is a multifactorial chronic disease. While important progress has been made to treat patients, many questions regarding aspects of this disease relating to carcinogenesis are still open. During carcinogenesis, cells exhibit cholesterol homeostasis deregulation. This results in an accumulation of intracellular cholesterol, which is required to sustain their high growth rate. Cholesterol efflux and influx are two metabolic pathways that are necessary to prevent cholesterol accumulation in the cells. Liver X receptors (LXRs) are nuclear receptors that, upon activation, induce the expression of ABC transporters, responsible for promoting cholesterol efflux, and the expression of IDOL (inducible degrader of low-density lipoprotein receptor), in charge of reducing cholesterol influx. Oxysterols, oxygenated derivatives of cholesterol formed through different pathways, have been discovered as LXR-specific ligands. Some oxysterols are involved in tumor formation while others are considered anti-tumor agents. In the present review, we discuss the involvement of cholesterol, oxysterols and LXRs in breast cancer pathophysiology, with an emphasis on the biological effects of LXR ligands.

## 1. Introduction

Breast cancer is a multifactorial chronic disease. It is now recognized that there are correlations between obesity and metabolic syndrome and the risk of developing breast cancer. It was also shown that circulating levels of Estrogen Receptor ER and estrogens were associated with adiposity and breast cancer. High blood cholesterol is common in obesity and metabolic syndrome [1], and its impact as a risk factor for breast cancer is controversial. Discrepancies between results may be explained by the distribution of blood cholesterol among the different major classes of lipoproteins (VLDL, LDL and HDL) and its modulation by lifestyle and menopausal status [2].

Both animal and human studies have shown that circulating levels of cholesterol closely mirror those of the primary metabolite of cholesterol, the oxysterol 27OHC, and that hypercholesterolemia results in high levels of 27OHC [3,4,5,6]. Several studies have demonstrated that this oxysterol functions as a mitogen in ER-positive tumors and as a ligand of the nuclear receptors liver X receptors (LXRs) [7]. LXRs (LXRα (also called NR1H3) and LXRβ (NR1H2)) are transcription factors that regulate the expression of key genes that are involved in lipid and cholesterol metabolism.

This evidence led researchers to study LXRs and their ligands (oxysterols and synthetic ligands) in relation to their involvement in breast tumorigenesis. The results of various studies showed that not all oxysterols derived from cholesterol act in the same manner as 27OHC. Indeed, synthetic and natural ligands of LXRs, e.g., T0901317 and 22(R)-hydroxycholesterol (22(R)-OHC), both suppressed proliferation and induced apoptosis in a breast cancer model cell line (ER+) [8]. Moreover, the activation of LXRs by T0901317 decreased the expression of Flotillin-2, a biomarker of lipid rafts, which play important roles in cancer progression and the Akt signaling pathway in the MCF-7 cell line [9]. 22(R)-OHC and 24(S)-hydroxycholesterol suppressed the proliferation of prostate and breast cancer cells [10]. Cancer cell lines with higher LXRα mRNA expression were more sensitive to 22(R)-OHC-induced inhibition [11].

**Figure 1:**
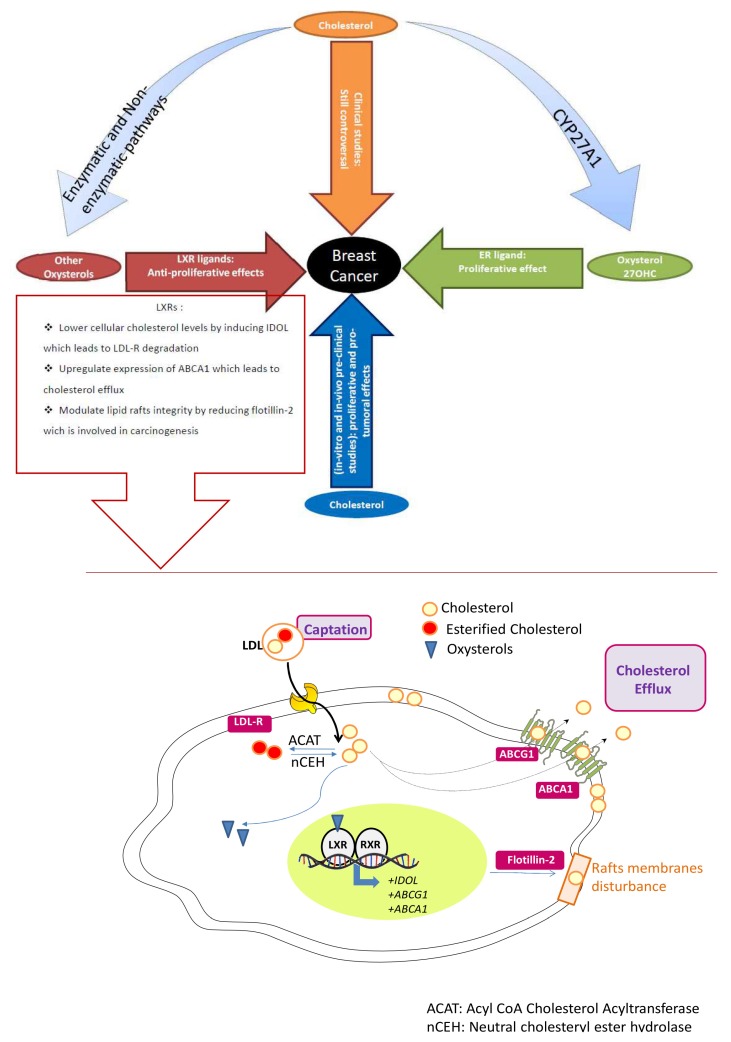
Influence of cholesterol, oxysterols and liver X receptors (LXRs) on breast cancer pathophysiology.

In this review, we have focused on the relationships between cholesterol, oxysterols, LXRs and breast cancer (Figure 1).

## 2. Cholesterol, Oxysterols, LXRs and Breast Cancer

All cells, including mammary cells, are able to synthesize cholesterol through the mevalonate pathway by an enzyme cascade in which HMG-CoA-reductase (HMGCR) plays a central role. Cells can also acquire cholesterol through lipoproteins. Indeed, lipoproteins mediate the delivery of cholesterol (from diet and biosynthesis) to cells from the blood stream.

Cholesterol is not only important as a component of cell membranes. It also serves as a precursor for steroid hormones, bile acids, vitamin D and oxysterols, and is a critical molecule for cell growth and function. [7,12,13,14,15].

Intracellular cholesterol is finely regulated by different complex mechanisms. A large number of experimental studies have shown that cancer cells exhibit deregulated transcriptional levels of several genes involved in cholesterol regulation and metabolism such as low-density lipoprotein receptor (*LDLR*), HMG-CoA reductase (*HMGCR*) and sterol regulatory element-binding protein (*SREBPS*) [16,17]. Indeed, many cancer cells show elevated LDL receptor levels and increased LDL uptake [18,19]. In a breast cancer cell model known for aggressive cell behavior (MDA-MB-231), LDL receptor has been shown to be upregulated and LDL stimulates cell migration [20]. Scavenger receptor-BI (SR-BI) is also often overexpressed in tumors, and is considered to contribute to increasing HDL-cholesterol uptake in cancer cells [18,21]. In MDA-MB-231 cells, knockdown of SR-BI inhibits migration in vitro and tumor growth in vivo [22]. Moreover, studies of cancer cells revealed that cholesterol biosynthesis, mediated by HMG-CoA reductase, is enhanced due to increased transcriptional regulation (mediated by SREBP-2) [23,24]. The effect of statins, hypocholesterolemic drugs that selectively inhibit HMG-CoA reductase, was also of interest to investigators. They have been shown to exhibit anti-proliferative and pro-apoptotic effects in numerous experimental studies [25,26]. Several mechanisms have been described for the effect of statins, among them inhibition of the generation of isoprenoids, which are necessary for the prenylation, addressing and localization of Ras proteins. The Ras superfamily of GTPases has well-established roles in cell proliferation, survival, migration, and invasion. In addition to this effect on cholesterol biosynthesis, another mechanism of action has been recently elucidated. The results of Bai et al. showed that simvastatin increased miR-140-5p in a dose-dependent manner via activating transcription factor NRF1, which reduced breast cancer (ER+ and ER−) cell proliferation and induced apoptosis [27]. The various observations and experiments using statins or genetic manipulation are evidence that the deregulation of cholesterol homeostasis leads to an accumulation of intracellular cholesterol, which is required to sustain the high growth rate and function of cancer cells observed in earlier studies [28,29].

In addition to the genes mentioned below, the transcription factors liver X receptors (LXRs) are also involved in maintaining intracellular cholesterol homeostasis by controlling its efflux and/or influx.

Some studies reported that increased cholesterol efflux and/or decreased influx are associated with decreased cancer cell proliferation and tumorigenesis. Indeed, El Roz et al. have demonstrated that LXR activation by TO901317 or 22ROHC in MCF-7 cells deprives cells of cholesterol by stimulating its efflux through the expression of ABCG1. This results in the inhibition of cell proliferation and in the stimulation of apoptosis [8]. In regard to the mechanism of action of cholesterol influx reduction, LXR inhibits LDL receptor expression through the induction of an E3 ligase that ubiquinates LDLRs called IDOL (inducible degrader of LDLR) [30].

### 2.1. Cholesterol and Breast Cancer in Animal Studies

A study by Llaverias et al. examined the role of plasma cholesterol in the development of mammary tumors and metastasis in an MMTV-PYMT mouse model of mammary cancer. Their data show that increased dietary intake of cholesterol alone (through a high cholesterol diet) results in significantly decreased tumor latency and increased tumor growth, supporting the idea that cholesterol itself can impact tumor pathophysiology. In the same study, it was demonstrated that in mammary tumors, increased expression of cyclin D1, a marker of tumor formation, is associated with cholesterol plasma levels. Moreover, increased expressions of SR-BI (scavenger receptor-BI) and LDL receptors in tumors were shown [16].

To explore the mechanism involved in LDL favoring breast cancer growth and invasiveness, dos Santos et al. analyzed the effects of LDL in different models of breast cancer. It was found that LDL (but not HDL) promotes cell proliferation, migration and loss of adhesion. Using in vivo models (on a high cholesterol diet), they also showed that breast tumors are consistently larger and more proliferative in hypercholesterolemic mice, and these mice also have increased lung metastasis. They revealed an overexpression of the survival pathways Akt and ERK and decreased adhesion molecules in breast cancer cells exposed to LDL [31].

The inhibition of PCSK9 (protein convertase subtilisin/kexin 9) is an efficient strategy for lowering cholesterol and low-density lipoprotein cholesterol (LDL-C) [32]. In a study by Momtazi-Borojeni et al., the anti-PCSK9 antibody was induced via a vaccine in mice inoculated with breast carcinoma cells. It was demonstrated that the rate of tumor growth was decreased by 21% in the vaccine group compared with the control group. In the same study, lifespan was increased by 4.2% in the vaccine group compared with the control group [33].

### 2.2. Cholesterol and Breast Cancer in Human Studies

Studies in humans on the associations between serum cholesterol and its carriers, lipoproteins, and breast cancer led to conflicting results. Some authors found a protective effect, others concluded that cholesterol is a risk factor, and some found no effect. Indeed, Chang et al. demonstrated that higher levels of VLDL-C and lower levels of ApoAI, a component of HDL, were significantly associated with breast cancer [34].

Another study suggests that higher levels of HDL-C may reduce breast cancer risk among premenopausal women [35].

The results of the ARIC cohort study suggest that low HDL-cholesterol among pre-menopausal women may be a marker of increased breast cancer risk [36].

Recent analyses revealed that breast cancer risk increased 1.6-fold in patients with hyperlipidemia [37]. In another study, it was demonstrated that patients with established breast cancer had higher LDL-C and lower VLDL-C, although no association between HDL or total cholesterol and breast cancer was obvious [38].

The meta-analysis realized by Touvier et al. confirmed the evidence of a modest but statistically significant inverse association between total cholesterol and more specifically HDL-C and the risk of breast cancer [39].

The British study ACALM (Algorithm for Comorbidities, Associations, Length of Stay and Mortality) showed that women aged 40 and over with high cholesterol were less likely to develop breast cancer by 45%. The risk of death in patients who developed cancer was reduced by 40% in patients with high cholesterol levels [40].

Since serum cholesterol is influenced by diet, especially by lipid consumption, and by hypocholesterolemic drugs, the effect of dietary cholesterol and statins on the incidence of breast cancer has also interested researchers.

In a population-based study, Hu et al. found that dietary cholesterol was positively associated with breast cancer risk, mainly in postmenopausal women [41].

In a meta-analysis conducted by Li et al., which examined the association between dietary cholesterol and breast cancer, the authors found that the association became significant when the consumption was greater than 0.37 g/day [42].

The findings of some meta-analyses do not support the hypothesis that statins have a protective effect against breast cancer [5,43,44]. However, in another meta-analysis, statin use was associated with reduced breast cancer recurrence [45].

From these studies, it is not clear whether total cholesterol makes the best risk biomarker. It was subsequently suggested that 27 OH could be a more suitable candidate.

## 3. 27OHC, Oxysterols, LXRs and Breast Cancer

Oxysterols are metabolites of cholesterol. Some of them are oxygenated enzymatically (e.g., 25-hydroxycholesterol (25OHC), 27-hydroxycholesterol (27OHC) and 24S-hydroxychlesterol (24OHC)), while some are not produced enzymatically (e.g., 7alpha/beta-hydroperoxycholesterol (7OOHC), 7 ketocholesterol (7KC)) [12,46].

Most oxysterols are described as LXR ligands. Ligand activation of LXRs leads to the recruitment of specific coactivators (e.g., steroid receptor coactivator-1 (SRC-1), PPARg coactivator-1alpha (PGC-1alpha), and activating signal cointegrator-2 (ASC-2)), which results in the transcription of target genes [47].

The oxysterol 27OHC is a primary metabolite of cholesterol synthesized by CYP27A1 and circulates at slightly higher concentrations compared to other oxysterols [48]. Based on many studies, 27OHC is considered as an LXR ligand and as an SERM produced endogenously [49] Therefore, it may be that 27OHC has different effects on cell growth, depending on the ER status. Indeed, it has been demonstrated that 27OHC stimulates proliferation in ER-positive breast cancer cell models [50]. Moreover, it has been shown that the exogenous co-administration of ER antagonists and 27OHC reverses the growth of breast tumors in a xenografted animal model in comparison with the administration of 27OHC alone [51]. In another study using LXR-/- breast cancer cells, 27OHC enhanced the induction of ER target genes. In contrast, 27OHC resulted in the upregulation of LXR target genes in ER-knocked down cells [52].

Moreover, the observation that elevated levels of CYP7B1, a cytochrome p450 enzyme responsible for the catabolism of 27OHC, are associated with better survival outcomes in mice suggests the involvement of this oxysterol in breast cancer pathophysiology [51]. Nevertheless, this effect may vary with the ER status of the tumor.

Considering the role of 27OHC in the growth of breast tumors, several investigators have conducted studies on tumors and in humans. The study of Wu et al. showed that intra-tumor levels of 27OHC are six-fold higher in ER-positive breast tumors in comparison with adjacent normal tissue [53]. Solheim et al. analyzed side chain oxysterols in breast cancer tumors (ER+ and ER−). They observed a correlation between esterified and free 27-hydroxycholesterol in both tumors. However, no significant differences in levels of other endogenous oxysterols were observed between ER+ and ER− tumors [54].

Exosomes from cancer cells are rich sources of biomarkers and may serve as useful for diagnosis. A study by Roberg-Larsen et al. found an increased level of 27OHC in exosomes from the MCF-7 (ER+) breast cancer cell line compared to exosomes derived from an estrogen receptor (ER−) breast cancer cell line (MDA-MB-231) and other control exosomes (from a non-cancerous cell line (HEK293) and human plasma) [55].

Using the European Prospective Investigation into Cancer and Nutrition (EPIC) Heidelberg cohort, Lu et al. analyzed the association between 27OHC and breast cancer risk. Their study showed that the association between serum 27OHC and breast cancer risk differs by menopausal status but not by age at diagnosis. A lack of association among premenopausal women at blood collection was observed. However, among postmenopausal women, higher serum 27OHC levels were associated with lower breast cancer risk [56].

Another oxysterol, 25OHC, has been found to be elevated in the circulation of breast cancer patients who have relapsed compared to those with primary disease [57].

Since the LXR agonist 27OHC is considered to influence breast cancer cell proliferation, studies have been conducted on the expression of LXR itself and oxysterol determination in human tumors and in cell lines. Hutchinson et al. evaluated the differences in oxysterols and LXR signaling between ER− and ER+ breast cancers and cell lines. They observed that ER− breast cancer cells are more responsive than ER+ breast cancer cells to LXR agonists. In the same study, they questioned whether the increased transcriptional activity of LXR found in cellular models (ER−) could be encountered in primary breast tumors. To verify this, they examined whether the expression of LXRalpha or LXRbeta was correlated with the expression of the LXR canonical target genes (ABCA1 and ApoE) in primary tumors with different ER statuses (ER− and ER+). They showed that ABCA1 correlated with LXRalpha in ER− but not in ER+ tumors. ApoE correlated with LXRalpha in both subtypes, but the correlation was much weaker in ER+ than in ER− tumors. Both ABCA1 and ApoE were assessed for their correlation with LXRbeta. ApoE weakly correlated with LXRbeta in ER+ tumors but was not correlated with LXR in ER-negative tumors; ABCA1 was not correlated with LXRbeta in either tumor type. From their observations, they concluded that ER status was inversely associated with the ability of LXR to induce canonical target gene expression [58].

The transcriptional activity of LXR (alpha and beta) is regulated by the corepressors NCOR1, NCOR2 and LCOR [59,60]. It is known that LXRalpha has a 100-fold higher binding affinity than LXRbeta for the corepressors NCOR1 and NCOR2 [61]. Based on these observations, it appears that the single measurement of LXR agonists is not sufficient; it seems necessary, for example, to determine both ligand concentrations and transcriptional activity. In the study of Hutchinson et al., it was demonstrated that MDA-MB-468 breast cancer cells (ER−) expressed significantly fewer NCOR1, NCOR2 and LCOR transcripts than the breast cancer cell line MCF7 (ER+). They also showed that the knock-down of these co-repressors equalized sensitivity to ligands between ER subtypes [58]. In another study, Pan et al. assessed the expression levels of ABCA1, ABCG1 and LXRbeta in triple negative breast cancer (TNBC) tissues and in non-cancerous mammary tissues. They identified that only ABCA1 in TNBC tissues was higher than that in non-cancerous mammary tissues. A high expression of ABCA1 in TNBC tissues was significantly associated with histological grade. However, no significant differences were identified between the expression levels of LXR-β and ABCG1 in both mammary tissues [62]. A mass spectrometry-based label-free quantification followed by functional annotation was performed to investigate the most significant deregulated proteins among various tissues from patients diagnosed with invasive ductal carcinoma: the primary breast tumor, axillary metastatic lymph nodes, and the corresponding contralateral and adjacent non-tumor tissues.. In this study, the differentially expressed proteins between the malignant and non-tumor breast tissues were mostly related to the LXR/RXR pathway [63].

In a recent study conducted to explain why triple negative breast cancer (TNBC) is more aggressive, specifically in African and African-American women, the authors compared the proteomic profile of this cancer (TNBC) with luminal A (LA) cancer in African-American (AA) and European-American (EA) patients. They demonstrated that representations of LXR/RXR signaling pathways were increased in LA samples from AA women and that this pathway was altered in TNBC samples from AA women. Furthermore, they showed that Cyp7B1, the enzyme responsible for the degradation of 27OHC, had strong immunoreactivity in the tumor cells of LA patients and less in TNBC tissues [64].

## 4. Conclusions

In summary, the influence of cholesterol on the incidence of breast cancer is still an exciting and controversial debate. On one hand, 27OHC, its primary oxysterol metabolite, known as an LXR ligand and an SERM, has been demonstrated to function as a mitogen and is now increasingly considered a potential risk biomarker. However, other oxysterols are considered anti-tumor agents. In addition, the LXR/RXR signaling pathway and Cyp7B1, the enzyme responsible for the degradation of 27OHC, are significantly increased in women with luminal A cancer compared to those with TNBC cancer. Furthermore, LXRalpha and LXRbeta and their co-repressors are differentially expressed in tumors with different ER statuses. These observations reinforce the importance of studying oxysterols and nuclear LXR receptors in breast cancer. The results of these studies would be helpful in conceiving new strategies for therapeutic intervention.

## References

[1] Must A., Spadano J., Coakley E.H., Field A.E., Colditz G., Dietz W.H. (1999). The disease burden associated with overweight and obesity. JAMA.

[2] Law M.R., Thompson S.G. (1991). Low serum cholesterol and the risk of cancer: An analysis of the published prospective studies. Cancer Causes Control..

[3] Borgquist S., Bjarnadottir O., Kimbung S., Ahern T.P. (2018). Statins: A role in breast cancer therapy?. J. Int. Med..

[4] Kimbung S., Markholm I., Bjöhle J., Lekberg T., von Wachenfeldt A., Azavedo E., Saracco A., Hellström M., Veerla S., Paquet E. (2018). Assessment of early response biomarkers in relation to long-term survival in patients with HER2-negative breast cancer receiving neoadjuvant chemotherapy plus bevacizumab: Results from the Phase II PROMIX trial. Int. J. Cancer.

[5] Garcia-Estevez L., Moreno-Bueno G. (2019). Updating the role of obesity and cholesterol in breast cancer. Breast Cancer Res..

[6] Karuna R., Holleboom A.G., Motazacker M.M., Kuivenhoven J.A., Frikke-Schmidt R., Tybjaerg-Hansen A., Georgopoulos S., van Eck M., van Berkel T.J., von Eckardstein A. (2011). Plasma levels of 27-hydroxycholesterol in humans and mice with monogenic disturbances of high density lipoprotein metabolism. Atherosclerosis.

[7] Umetani M., Domoto H., Gormley A.K., Yuhanna I.S., Cummins C.L., Javitt N.B., Korach K.S., Shaul P.W., Mangelsdorf D.J. (2007). 27-Hydroxycholesterol is an endogenous SERM that inhibits the cardiovascular effects of estrogen. Nat. Med..

[8] El Roz A., Bard J.M., Huvelin J.M., Nazih H. (2012). LXR agonists and ABCG1-dependent cholesterol efflux in MCF-7 breast cancer cells: Relation to proliferation and apoptosis. Anticancer Res..

[9] Carbonnelle D., Luu T.H., Chaillou C., Huvelin J.M., Bard J.M., Nazih H. (2017). LXR Activation Down-regulates Lipid Raft Markers FLOT2 and DHHC5 in MCF-7 Breast Cancer Cells. Anticancer Res..

[10] Fukuchi J., Kokontis J.M., Hiipakka R.A., Chuu C.P., Liao S. (2004). Antiproliferative effect of liver X receptor agonists on LNCaP human prostate cancer cells. Cancer Res..

[11] Chuu C.-P., Lin H.-P. (2010). Antiproliferative Effect of LXR Agonists T0901317 and 22(R)-Hydroxycholesterol on Multiple Human Cancer Cell Lines. Anticancer Res..

[12] Janowski B.A., Willy P.J., Devi T.R., Falck J.R., Mangelsdorf D.J. (1996). An oxysterol signalling pathway mediated by the nuclear receptor LXR alpha. Nature.

[13] Wei W., Schwaid A.G., Wang X., Wang X., Chen S., Chu Q., Saghatelian A., Wan Y. (2016). Ligand Activation of ERRalpha by Cholesterol Mediates Statin and Bisphosphonate Effects. Cell Metab..

[14] Ahmad F., Sun Q., Patel D., Stommel J.M. (2019). Cholesterol Metabolism: A Potential Therapeutic Target in Glioblastoma. Cancers.

[15] Simons K., Ikonen E. (2000). How cells handle cholesterol. Science.

[16] Llaverias G., Danilo C., Mercier I., Daumer K., Capozza F., Williams T.M., Sotgia F., Lisanti M.P., Frank P.G. (2011). Role of cholesterol in the development and progression of breast cancer. Am. J. Pathol..

[17] Scheinman E.J., Rostoker R., Leroith D. (2013). Cholesterol affects gene expression of the Jun family in colon carcinoma cells using different signaling pathways. Mol. Cell Endocrinol..

[18] Hoque M., Rentero C., Conway J.R., Murray R.Z., Timpson P., Enrich C., Grewal T. (2015). The cross-talk of LDL-cholesterol with cell motility: Insights fromhe Niemann Pick Type C1 mutation and altered integrin trafficking. Cell Adhes. Migr..

[19] Tatidis L., Masquelier M., Vitols S. (2002). Elevated uptake of low density lipoprotein by drug resistant human leukemic cell lines. Biochem. Pharmacol..

[20] Antalis C.J., Uchida A., Buhman K.K., Siddiqui R.A. (2011). Migration of MDA-MB-231 breast cancer cells depends on the availability of exogenous lipids and cholesterol esterification. Breast Cancer Res. Treat..

[21] Cruz P.M., Mo H., McConathy W.J., Sabnis N., Lacko A.G. (2013). The role of cholesterol metabolism and cholesterol transport in carcinogenesis: A review of scientific findings, relevant to future cancer therapeutics. Front. Pharmacol..

[22] Danilo C., Gutierrez-Pajares J.L., Mainieri M.A., Mercier I., Lisanti M.P., Frank P.G. (2013). Scavenger receptor class B type I regulates cellular cholesterol metabolism and cell signaling associated with breast cancer development. Breast Cancer Res..

[23] Clendening J.W., Penn L.Z. (2012). Targeting tumor cell metabolism with statins. Oncogene.

[24] Krycer J.R., Phan L. (2012). A key regulator of cholesterol homeostasis, SREBP-2, can be targeted in prostate cancer cells with natural products. Biochem. J..

[25] Huang S.W., Chyuan I.T., Shiue C., Yu M.C., Hsu Y.F., Hsu M.J. (2020). Lovastatin-mediated MCF-7 cancer cell death involves LKB1-AMPK-p38MAPK-p53-survivin signalling cascade. J. Cell Mol. Med..

[26] Shibata M.A., Kavanaugh C., Shibata E., Abe H., Nguyen P., Otsuki Y., Trepel J.B., Green J.E. (2003). Comparative effects of lovastatin on mammary and prostate oncogenesis in transgenic mouse models. Carcinogenesis.

[27] Bai F., Yu Z., Gao X., Gong J., Fan L., Liu F. (2019). Simvastatin induces breast cancer cell death through oxidative stress up-regulating miR-140-5p. Aging.

[28] Clayman R.V., Gonzalez R., Elliott A.Y., Gleason D.E., Dempsey M.E. (1983). Cholesterol accumulation in hetero transplanted renal cell cancer. J. Urol..

[29] Swyer G. (1942). The cholesterol content of normal and enlarged prostates. Cancer Res..

[30] Zelcer N., Hong C., Boyadjian R., Tontonoz P. (2009). LXR regulates cholesterol uptake through Idol dependent ubiquitination of the LDL receptor. Science.

[31] Dos Santo C.R., Domingues G., Matias I., Matos J., Fonseca I., de Almeida J.M., Dias S. (2014). LDL-Cholesterol signling induces breast cancer proliferation and invasion. Lipids Health Dis..

[32] Jaworsky K., Jankowsky P., Kosior D.A. (2017). PCSK9 inhibitors-From discovery of a single mutation to a groundbreaking therapy of lipid disorders in one decade. Arch. Med. Sci..

[33] Momtazi-Borojeni A.A., Nik M.E., Jaafari M.R., Banach M., Sahebkar A. (2019). Effects of immunization against PCSK9 in an experimental model of breast cancer. Arch. Med. Sci..

[34] Chang S.J., Hou M.F., Tsai S.M., Wu S.H., Hou L.A., Ma H., Shann T.Y., Wu S.H., Tsai L.Y. (2007). The association between lipid profiles and breast cancer among Taiwanese women. Clin. Chem. Lab. Med..

[35] Kim Y., Park S.K., Han W., Kim D.H., Hong Y.C., Ha E.H., Ahn S.H., Noh D.Y., Kang D., Yoo K.Y. (2009). Serum high-density lipoprotein cholesterol and breast cancer risk by menopausal status, body mass index, and hormonal receptor in Korea. Cancer Epidemiol. Biomark. Prev..

[36] Kucharska-Newton A.M., Rosamond W.D., Mink P.J., Alberg A.J., Shahar E., Aaron R., Folsom A.R. (2008). HDL-cholesterol and incidence of breast cancer in the ARIC cohort study. Ann. Epidemiol..

[37] Potluri R., Lavu D., Uppal H., Chandran S. (2014). P740 Hyperlipidemia as a risk factor for breast cancer?. Cardiovasc. Res. Suppl..

[38] Laisupasin P., Thompat W., Sukarayodhin S., Sornprom A., Sudjaroen Y. (2013). Comparison of Serum lipid Profiles between normal controls and breast cancer patients. J. Lab. Physcians..

[39] Touvier M., Fassier P., His M., Norat T., Chan D.S., Blacher J., Hercberg S., Galan P., Druesne-Pecollo N., Latino-Martel P. (2015). Cholesterol and breast cancer risk: A systematic review and meta-analysis of prospective studies. Br. J. Nutr..

[40] Carter P.R., McGowan J., Uppal H., Chandran S., Sarma J., Potluri R. (2016). Hyperlipidemia reduces mortality in breast, prostate, lung and bowel cancer. Heart.

[41] Hu J., La Vecchia C., de Groh M., Negri E., Morrison H., Mery L. (2012). Canadian Cancer registries epidemiology research G: Dietary cholesterol intake and cancer. Ann. Oncol..

[42] Li C., Yang L., Zhang D., Jiang W. (2016). Systematic review and meta-analysis suggest that dietary cholesterol intake increases risk of breast cancer. Nutr. Res..

[43] Islam M.M., Yang H.C., Nguyen P.A., Poly T.N., Huang C.W., Kekade S., Khalfan A.M., Debnath T., Li Y.J., Abdul S.S. (2017). Exploring association between statin use and breast cancer risk: An updated meta-analysis. Arch. Gynecol. Obstet..

[44] Undela K., Srikanth V., Bansal D. (2012). Statin use and risk of breast cancer: A meta-analysis of observational studies. Breast Cancer Res. Treat..

[45] Mansourian M., Haghjooy-Javanmard S., Eshraghi A., Vaseghi G., Hayatshahi A., Thomas J. (2016). Statins Use and Risk of Breast Cancer Recurrence and Death: A Systematic Review and Meta-Analysis of Observational Studies. J. Pharm. Pharm. Sci..

[46] Bovenga F., Sabbà C., Moschetta A. (2015). Uncoupling nuclear receptor LXR and cholesterol metabolism in cancer. Cell Metab..

[47] Cannon M.V., van Gilst W.H., de Boer R.A. (2016). Emerging role of liver X receptors in cardiac pathophysiology and heart failure. Basic Res. Cardiol..

[48] Prunet C., Petit J.M., Ecarnot-Laubriet A., Athias A., Miguet-Alfonsi C., Rohmer J.F., Steinmetz E., Neel D., Gambert P., Lizard G. (2006). High circulating levels of 7beta- and 7alpha-hydroxycholesterol and presence of apoptotic and oxidative markers in arterial lesions of normocholesterolemic atherosclerotic patients undergoing endarterectomy. Pathol. Biol. (Paris).

[49] Nelson E.R. (2018). The significance of cholesterol and its metabolite, 27-hydroxycholesterol in breast cancer. Mol. Cell Endocrinol..

[50] DuSell C.D., Umetani M., Shaul W., Mangelsdorf D.J., McDonnell D.P. (2008). 27-hydroxycholesterol is an endogenous selective estrogen receptor modulator. Mol. Endocrinol..

[51] Nelson E.R., Wardell S.E., Jasper J.S., Park S., Suchindran S., Howe M.K., Carver N.J., Pillai R.V., Sullivan P.M., Sondhi V. (2013). 27-Hydroxycholesterol links hypercholesterolemia and breast cancer pathophysiology. Science.

[52] Nelson E.R., DuSell C.D., Wang X., Howe M.K., Evans G., Michalek R.D., Umetani M., Rathmell J.C., Khosla S., Gesty-Palmer D. (2011). The oxysterol, 27-hydroxycholesterol, links cholesterol metabolism to bone homeostasis through its actions on the estrogen and liver X receptors. Endocrinology.

[53] Wu Q., Ishikawa T., Sirianni R., Tang H., McDonald J.G., Yuhanna I.S., Thompson B., Girard L., Mineo C., Brekken R.A. (2013). 27-hydroxycholesterol promotes cell autonomous, ER-positive breast cancer growth. Cell Rep..

[54] Solheim S., Hutchinsonb S.A., Lundanesa E., Wilsona S.R., James L., Thorneb J.L., Roberg-Larsena H. (2019). Fast liquid chromatography-mass spectrometry reveals side chain oxysterol heterogeneity in breast cancer tumour samples. J. Steroid. Biochem. Mol. Biol..

[55] Roberg-Larsen H., Lund K., Seterdal K.E., Solheim S., Vehus T., Solberg N., Krauss S., Lundanes E., Wilson S.R. (2017). Mass spectrometric detection of 27-hydroxycholesterol in breast cancer exosomes. J. Steroid. Biochem. Mol. Biol..

[56] Lu D.L., Le Cornet C., Sookthai D., Johnson T.S., Kaaks R., Fortner R.T. (2019). Circulating 27-hydroxycholesterol and breast cancer risk: Results from the EPICHeidelberg Cohort. J. Natl. Cancer Inst..

[57] Dalenc F., Iuliano L., Filleron T., Zerbinati C., Voisin M., Arellano C., Chatelut E., Marquet P., Samadi M., Roche H. (2016). Circulating oxysterol metabolites as potential new surrogate markers in patients with hormone receptor-positive breast cancer: Results of the OXYTAM study. J. Steroid. Biochem. Mol. Biol..

[58] Hutchinson S.A., Lianto P., Roberg-Larsen H., Battaglia S., Hughes T.A., Thorne J.L. (2019). ER-Negative breast cancer is highly responsive to cholesterol metabolite signalling. Nutrients.

[59] Doig C.L., Singh P.K., Dhiman V.K., Thorne J.L., Battaglia S., Sobolewski M., Maguire O., O’Neill L.P., Turner B.M., McCabe C.J. (2012). Recruitment of NCOR1 to VDR target genes is enhanced in prostate cancer cells and associates with altered DNA methylation patterns. Carcinogenesis.

[60] Jalaguier S., Teyssier C., Nait Achour T., Lucas A., Bonnet S., Rodriguez C., Elarouci N., Lapierre M., Cavailles V. (2017). Complex regulation of LCoR signaling in breast cancer cells. Oncogene.

[61] Hu X., Li S., Wu J., Xia C., Lala D.S. (2003). Liver X receptors interact with corepressors to regulate gene expression. Mol. Endocrinol..

[62] Pan H., Zheng Y., Pan Q., Chen H., Chen F., Wu J., Di D. (2019). Expression of LXR-β, ABCA1 and ABCG1 in human triple-negative breast cancer tissues. Oncol. Rep..

[63] Gomig T.H.B., Cavalli I.J., Souza R.L.R., Vieira E., Lucena A.C.R., Batista M., Machado K.C., Marchini F.K., Marchi F.A., Lima R.S. (2019). Quantitative label-free mass spectrometry using contralateral and adjacent breast tissues reveal differentially expressed proteins and their predicted impacts on pathways and cellular functions in breast cancer. J. Proteomics..

[64] Torres-Luquis O., Madden K., N’dri N.M., Berg R., Olopade O., Ngwa W., Abuidris D., Mittal S., Lyn-Cook B., Mohammed S. (2019). LXR/RXR pathway signaling associated with triple-negative breast cancer in African American Women. Breast Cancer.

